# Zinc solubilization characteristics of efficient siderophore-producing soil bacteria

**Published:** 2019-10

**Authors:** Ebrahim Eshaghi, Rahim Nosrati, Parviz Owlia, Mohammad Ali Malboobi, Pejman Ghaseminejad, Mohammad Reza Ganjali

**Affiliations:** 1 Department of Biology, Nourdanesh Institute of Higher Education, Meymeh, Isfahan, Iran; 2 Cellular and Molecular Research Center, School of Medicine, Guilan University of Medical Sciences, Rasht, Iran; 3 Department of Pharmaceutical Biotechnology, School of Pharmacy, Mashhad University of Medical Sciences, Mashhad, Iran; 4 Molecular Microbiology Research Center, Shahed University, Tehran, Iran; 5 Department of Plant Biotechnology, National Institute of Genetic Engineering and Biotechnology, Tehran, Iran; 6 Department of Research and Development, Green Biotech Inc, Tehran, Iran; 7 Center of Excellence in Electrochemistry, Faculty of Chemistry, University of Tehran, Tehran, Iran

**Keywords:** Rhizobacteria, Siderophore, Zinc solubilization, Plant growth-promoting rhizobacteria (PGPR), *Pseudomonas japonica*

## Abstract

**Background and Objectives:**

Iron and zinc are two essential micro-nutrients for plant growth and development. Therefore, isolation of siderophores-producing and zinc-solubilizing rhizobacteria involved in bio-availability of these elements is of great interest.

**Materials and Methods:**

In this study, soil samples collected from slightly alkaline soil types were screened for high levels of siderophore secretion and zinc solubilization.

**Results:**

Among positive colonies, three isolates, named F21A, F37 and F38, were able to secrete siderophore at high levels, ranged between 200 and 300 μM/liter. A close association was observed between siderophore production capability and growth rate as an indicator of active metabolism. Siderophore production was closely correlated with the level of zinc ion released into the medium as well. All three siderophore producing isolates were able to withstand temperature as high as 37°C, high concentration of NaCl (up to 2.5%) and a wide range of initial pH from 6 to 9 while hydrolyzing Zn compounds actively. One of the isolates, F21A, tolerated the presence of 200 mgl^−1^ of zinc. Biochemical and molecular characteristics are indicative that these isolates are *Pseudomonas japonica*. As experienced in a greenhouse experiment, inoculation with the F21A and F37 isolates significantly increase the plants height, fresh and dry weight of corn with compared to control.

**Conclusion:**

These findings demonstrated that the potential of *P. japonica* strains as plants growth promoting rhizobacteria (PGPR) in iron and zinc deficient soils.

## INTRODUCTION

Macro- and micro-nutrients are necessary for plant’s growth and reproduction. These nutrients are supplemented in inorganic or organic forms and taken up by the plant’s roots along with water. Bacteria play important role in mobilizing and absorption of nutrients for plants (1). In the recent decades, several bacterial strains, formulated as bio-fertilizers, have been introduced to improve NPK nutrition (2). However, few works have reported the role of bacteria in micronutrient absorption.

Iron as an essential micronutrient play important diverse roles in chlorophyll biosynthesis, redox reactions and various physiological activities (3). The availability of iron to plants particularly in alkaline soils is mostly limited (4). In iron-depleted conditions, microorganisms secreted siderophores which promote the process of iron uptake (5). Siderophores are low-molecular-weight organic compounds with high affinity and specificity for iron (6). Typically, siderophores are classified as catecholates, hydroxamates, and carboxylates types, depending on the chemical nature of their coordination sites with iron (6, 7). Siderophores promote the growth of plants via Fe uptake and subsequent increase of their yield (1).

Zinc (Zn) is also an essential element needed as a cofactor for the activities of several enzymes involved in the promotion of plant growth. Lack of Zn is one of the most common micronutrient deficiencies, particularly in soils with high pH (8). Globally, more than 30% of soils have low availability of Zn ion, which is the preferable form for plant uptake (9). Yet, about 96% to 99% of the exogenously applied Zn is converted into various insoluble forms within a few days after application, depending on the soil types and physicochemical reactions (10). In plants, Zn deficiency is manifested as a remarkable reduction in height and development of whitish brown patches that subsequently turn to necrotic spots (11). Zn solubilization can be accomplished by rhizobacteria through a range of mechanisms including excretion of metabolites such as organic acids, proton extrusion or production of chelating agents (12).

The aim of this study was to isolate beneficial bacteria which are capable of producing siderophores, and simultaneously, can effectively hydrolyze inorganic Zn compounds. The effects of several environmental conditions such as temperature, salt concentration, pH, and Zn concentration on performance of the selected isolates were assessed. Then, the capability of improving maize growth was assessed through zinc and iron availability to demonstrate the possible effects of these isolates on plant.

## MATERIALS AND METHODS

### Isolation of siderophore-producing and zinc-solubilizing bacteria

Eighty-five different soil samples were collected from the rhizosphere of different crops such as corn, sunflower, grapes, pistachio, alfalfa and vegetables (tomato, cabbage, potato, carrot, bean, lettuce and onion) from five regions of Iran (Tehran, Qom, Karaj, Kashan and Isfahan cities) in sterile tins and transferred to the laboratory. One gram from each sample was added into 100 ml distilled water and serial dilutions were prepared for streaking on selective solid medium. Siderophore producing bacteria was screened according to Alexander and Zuberer (13) method on Chrome azurol S (CAS) agar medium by streaking and incubation at 28°C for 2–5 days. The isolates with orange color surrounding the colony were purified. Screening for zinc solubilization was conducted by plating on mineral salts medium (MSM) supplemented with 0.1% of insoluble zinc oxide (ZnO) (14). The plates were incubated at 28°C for 3 days, and the colonies exhibiting clear zones were purified. The bacterial isolates solubilizing zinc ion on MSM agar were also screened for high siderophore producing activity and vice versa.

### Zinc solubilization and siderophore producing activity measurements

To examine the efficiency of Zn solubilization, 20 μl of the bacterial suspensions (~10^4^ CFUml^−1^) were spotted on the center of solid MSM containing insoluble ZnO and incubated at 28°C. The diameters of colonies and the halo zones surrounding the bacteria were measured after 2, 4 and 7 days in triplicates. Zn solubilizing index (ZSI) was calculated as the ratio of (halo+colony)/colony diameters (15).

To evaluate the siderophore producing activity, 20 μl of the bacterial suspensions (~10^4^ CFUml^−1^) were spotted on the center of CAS agar medium and incubated at 28°C. The diameters of colonies and orange color zones surrounding the colonies were measured after 2, 4 and 7 days in triplicates. Siderophore producing index (SPI) was calculated as the ratio of (colored zone+colony)/colony diameters (16). Siderophore typing was carried out using an overlay technique in which a modified CAS medium (O-CAS assay) was used as described by Pérez-Miranda et al. (7).

### Quantitative assay of siderophore production

One hundred μl of each bacterial suspension (~10^4^ CFUml^−1^) was inoculated into 100 ml Erlenmeyer flasks containing 40 ml of broth standard succinate medium (SSM/consisting of g L^−1^: K_2_HPO_4_, 6.0; KH_2_PO_4_, 3.0; MgSO_4_, 7H_2_O, 0.2; (NH_4_)_2_ SO_4_, 1.0; and succinic acid 4.0, pH 7.0.) and incubated at 28°C with constant shaking at 120 rpm. After 40 h of incubation, the media were centrifuged at 10,000 ×g for 10 min at room temperature and filtered through 0.22 μm membrane filter. The concentration of siderophore in cell free supernatants were estimated based on A=ɛBC formula described by Carrillo-Castaneda (17). Furthermore, the molecular weights of the extracted siderophores in the samples were determined using sodium dodecyl sulphate polyacrylamide gel electrophoresis (SDS-PAGE) with 12% separating and 4.0% stacking gel (18). The bands’ sizes were estimated by comparing to low range molecular weight marker proteins.

### The effects of growth conditions on siderophore production

In a series of time-coursed quantitative experiments, the effects of various growth conditions, including incubation temperature at 20, 28, and 37°C, 1, 2.5, and 5 percent NaOH (w/v) and pH 6 to 9, on the bacterial growth rate (the logarithm of CFUml^−1^) and siderophore production level secreted into the culture medium, were measured for the selected isolates, F21A, F37 and F38, while growing in broth SSM. In all assays, pH7, temperature of 28°C and lack of NaCl was used as control condition. Samplings were comprised of collection of 100 μl of medium at 0, 24, 40 and 72 h of incubations. Serial dilution was prepared and the growth rates were assay. The release of siderophores into the culture supernatant was measured using previously described method (17). Effects of different concentrations of Fe (0, 25, 50, 100 and 200 mM) and Zn (0, 75, 125, 250, and 500 mM) on the amount of secreted siderophores were measured for the selected isolates in broth SSM, in triplicates.

### Quantitative assessments of zinc solubilization

For the measurement of hydrolyzed soluble Zn, 100μl of selected bacterial suspension (~10^4^ CFUml^−1^) was inoculated into 250 ml Erlenmeyer flasks containing 100 ml of MSM medium supplemented with 0.1% insoluble zinc compounds and incubated at 28°C with constant shaking at 120 rpm. After 0, 2, 3, 4 and 7 days of incubation, 400 μl of samples were collected and centrifuged for 20 min at 2,000 rpm. Then, 10 μl of supernatant was used for released Zn assay by atomic absorption spectrophotometry (10). A non-inoculated medium was used as control. To measure the inhibitory effect of the available zinc on bacterial growth, the propagation rates were determined in nutrient broth containing different concentrations of soluble zinc using 25, 50, 100 and 200 mgl^−1^ of ZnSO_4_.

### Molecular identification of the selected isolates

Three selected isolates with the highest siderophore production were identified by physiological and biochemical tests, including colony morphology, the Gram staining, utilization of citrate and different sugars, H_2_S production, motility, as well as the activities of catalase, oxidase and urease as described in a previous work (19). *16S rDNA* amplification and sequencing were carried out using universal bacterial primers 27F; (AGAGTTTGATCCTGGCTCAG) and 1492R; (GGTTACCTTGTTACGACTT) to amplify a ~1.5 kb fragment (20). Sequence similarities were analyzed at NCBI GenBank database using BLAST program at https://blast.ncbi.nlm.nih.gov/Blast.cgi (20).

### Evaluation of PGPR efficiency of the selected strains at greenhouse assays

Pot culture experiments were conducted from August 2015 to October 2015 in 4 liter pots filled with a mixture of equal volumes of soil and sand. For inoculation, 2 ml of the bacterial suspension containing 10^7^–10^8^ CFU/ml (1×10^4^–10^5^ per gram soil) were used for the treatment of hybrid maize seeds (*Zea mays* L. CV. single-cross 704) and placed at the same depth in all pots. The control treatment consisted of water-treated seeds (without bacteria inoculation). The pots were arranged in a completely randomized block design with twenty-one treatments and three replicates. Treatments were consisted of two controls in which no bacteria were used; T1 with no Fe added and T2 with EDDHA-Fe 6% iron fertilizer; inoculation with individual bacterial strains, F21A, F21B, F37, F38, Z10, Z11 and Z29 in T3–T9; and double strain treatments with (F21A+ Z10), (F21B+ Z10), (F37+ Z10), (F38+ Z10), (F21A+ Z11), (F21B+ Z11), (F37+ Z11), (F38+ Z11), (F21A+ Z29), (F21B+ Z29), (F37+ Z29), T21 (F38+ Z29) into T10–T21, respectively. Plants were grown for 60 days under controlled greenhouse conditions: 25±5°C, day-night cycles of 16-8 h and 40–60 percent humidity and were daily watered with fresh water. Growth parameters, such as shoot length, wet and dry weights biomass of shoot were recorded.

### Statistical analysis

Statistical analyses were done using statistics software GraphPad Prism 8 (GraphPad Software, Inc.). Analyses were performed with using one-way analysis of variance (ANOVA) and Tukey’s test to compare the differences between the means (P < 0.05). Plant growth parameters were compared using ANOVA and Duncan’s means comparison.

## RESULTS

### Isolation of zinc solubilizing and siderophore producing bacteria

Out of 85 soil samples, 30 isolates with detectable zinc solubilizing trait and 23 isolates with high levels of siderophore production were isolated using MSM or CAS agar media, respectively. In a series of the subsequent re-examination experiments, 16 isolates capable of producing large halo-forming colonies on both MSM or CAS agar media, were selected for further investigation.

### Zinc solubilization and siderophore production assay

In plate-based assays, we found the largest zinc solubilizing clear zone diameter for Z16A and F24 (42 mm) whereas F74 isolate showed the smallest clear zone within 7 days. For siderophore secretion efficiency of the selected strains, the largest orange zone was observed for F37 (35 mm) followed by Z16A (34.5 mm) within 7 days. For better comparisons, the above data were standardized to ZSI and SPI indices as described in section 2.2. As shown in Table 1, the ZSI and SPI values were increased during the course of experiment for all the isolates. The highest ZSI value was observed for F37 (4.75), while the lowest level was for F74 (1.75), both in 7 days. The highest SPI value was observed for F37 (3.88) in 7 days while the lowest value was observed for F84 (1.07) in the same period of time. Typing of siderophores by overlay technique showed that 75 percent of isolates produced hydroxamate type and 25 percent of bacteria secreted carboxylate type, while none produced catecholate type of siderophore (Table 1).

**Table 1:** Zinc solubilization indexand siderophore properties of the selected isolates.

**Bacterial strains**	**Zn solubilizing index^a^**			**Siderophore production index^a^**			**Type of siderophoreb**	**Siderophore concentrationer**
	
	**2 days**	**4 days**	**7 days**	**2 days**	**4 days**	**7 days**		
F9	2.23 ± 0.02^d^	2 ± 0.0^i^	2.06 ± 0.0^g^	1.12 ± 0.05^f^	1.2 ± 0.05^i^	1.15 ± 0.03^ij^	A	94^c^
F14	2.62 ± 0.04^c^	2.55 ± 0.03^g^	3.33 ± 0.05^d^	1.62 ± 0.13^de^	1.9 ± 0.0^d^	2 ± 0.08^f^	B	58^e^
Z16A	3 ± 0.0^b^	2.66 ± 0.04^f^	2.62 ± 0.02^e^	1.62 ± 0.0^de^	1.22 ± 0.0^ghi^	1.17 ± 0.03^ij^	A	76^d^
Z20B	3.75 ± 0.05^a^	3.9 ± 0.05^b^	3.73 ± 0.07^c^	1.5 ± 0.02^e^	1.58 ± 0.02^ef^	1.58 ± 0.03^g^	A	46^f^
F21A	2.75 ± 0.06^bc^	3.62 ± 0.1^c^	3.75 ± 0.06^bc^	2.37 ± 0.02^c^	2.62 ± 0.1^c^	3.62 ± 0.06^b^	B	200^b^
F21B	2.62 ± 0.04^c^	3.62 ± 0.06^c^	3.75 ± 0.11^bc^	2.85 ± 0.05^b^	2.85 ± 0.05^b^	3.5 ± 0.15^c^	B	194^b^
F22	2.12 ± 0.01^de^	2.25 ± 0.04^h^	2.37 ± 0.04^ef^	1.71 ± 0.0^d^	1.66 ± 0.06^e^	1.6 ± 0.05^g^	A	32^gh^
F24	1.84 ± 0.02^e^	1.94 ± 0.0^i^	2.21 ± 0.02^fg^	1.12 ± 0.0^f^	1.2 ± 0.01^hi^	1.35 ± 0.02^h^	A	25^h^
F27	3.75 ± 0.11^a^	3.88 ± 0.1^b^	4 ± 0.05^b^	1.5 ± 0.06^e^	1.17 ± 0.03^fg^	1.64 ± 0.01^g^	B	90^c^
F33	2.77 ± 0.04^bc^	3.55 ± 0.08^c^	3.88 ± 0.1^bc^	0^g^	0^j^	1.08 ± 0.04^j^	A	40^fg^
F37	3.5 ± 0.9^a^	4.12 ± 0.18^a^	4.75 ± 0.0^a^	3.14 ± 0.08^a^	3.5 ± 0.09^a^	3.88 ± 0.1^a^	A	300^a^
F38	2.9 ± 0.0^bc^	3.18 ± 0.04^d^	3.63 ± 0.34^c^	2.42 ± 0.0^c^	2.75 ± 0.05^bc^	3.11 ± 0.06^d^	A	296.6^a^
F54	1.5 ± 0.03^f^	1.62 ± 0.0^j^	2.12 ± 0.12^fg^	1.14 ± 0.0^f^	1.14 ± 0.1^i^	1.25 ± 0.0h^i^	A	4.5^i^
F74	1.37 ± 0.04^f^	1.44 ± 0.02^k^	1.75 ± 0.01^h^	1.25 ± 0.04^f^	1.37 ± 0.04^gh^	1.33 ± 0.03^h^	A	3^i^
F83	3.5 ± 0.0^a^	3.62 ± 0.06^c^	4 ± 0.11^b^	1.5 ± 0.02^e^	2.11 ± 0.06^d^	2.22 ± 0.05^e^	A	1^i^
F84	3 ± 0.0^b^	3 ± 0.27^e^	3.88 ± 0.1^bc^	0^g^	0^j^	1.07 ± 0.01^j^	A	6.8^i^

a Data are presented as means ± standard errors of three replicates. Means comparison was done with Tukey’s test. Means with the same letters are ranked in the same groups.

b A: hydroxamate siderophore, B: carboxylate siderophore.

### Quantitative assay of siderophore production

Using SSM broth, a wide range of 0.8 to ~300 μMl^−1^ was obtained for selected isolates after 40 h. The isolate F37 showed the highest siderophore production (300 μM^−1^) in the liquid medium, followed by F38 and F21A with the activities of 297 and 200 μMl^−1^, respectively (Table 1). To confirm the siderophore production by these isolates, the SDS-PAGE analysis of the extracted siderophores samples was used, which revealed the presence of 18, 11, and 18 kD bands for F21A, F37 and F38, respectively.

### Assessments of siderophore production in various growth conditions

**Fig. 1:**
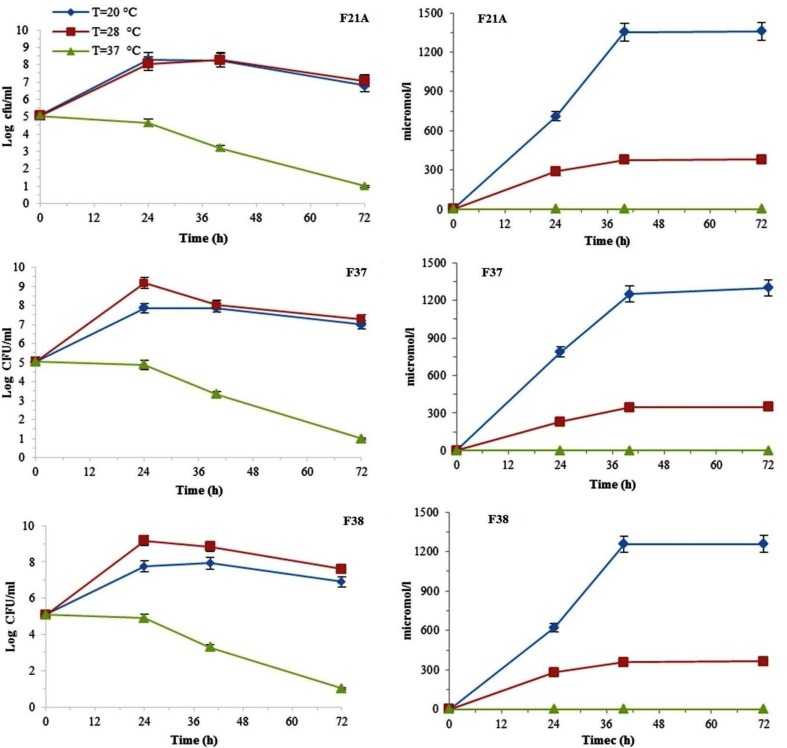
The effect of temperature on the bacterial growth kinetics (panels A, C and E) and siderophore production level (panels B, D and F) in liquid medium. All data presented as mean ± SEM of three replicates.

**Fig. 2:**
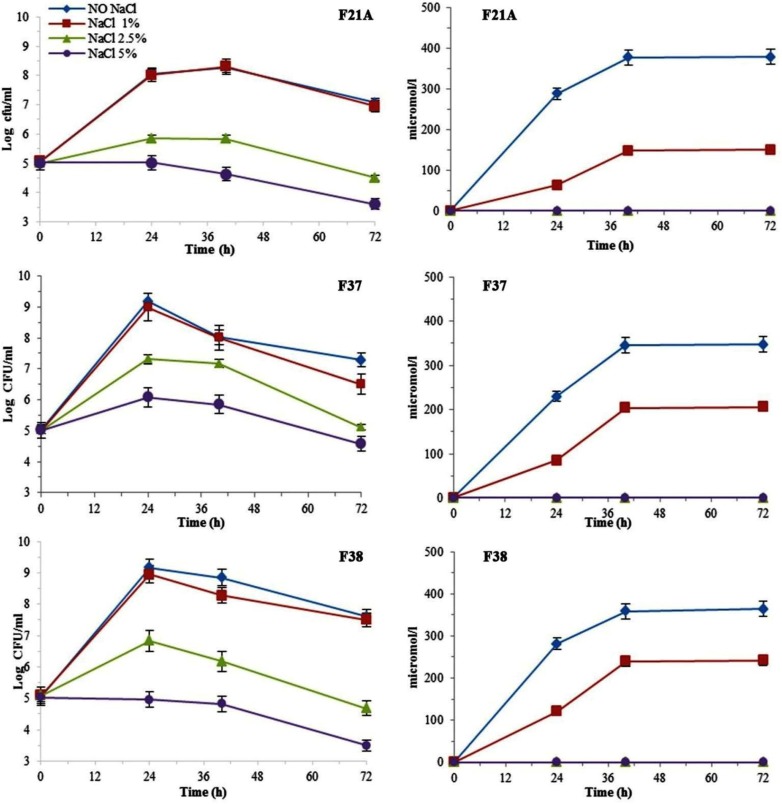
The effect of NaCl amounts added to the liquid medium on bacterial growth kinetics and siderophore production level. All data presented as mean ± SEM of three replicates.

**Fig. 3:**
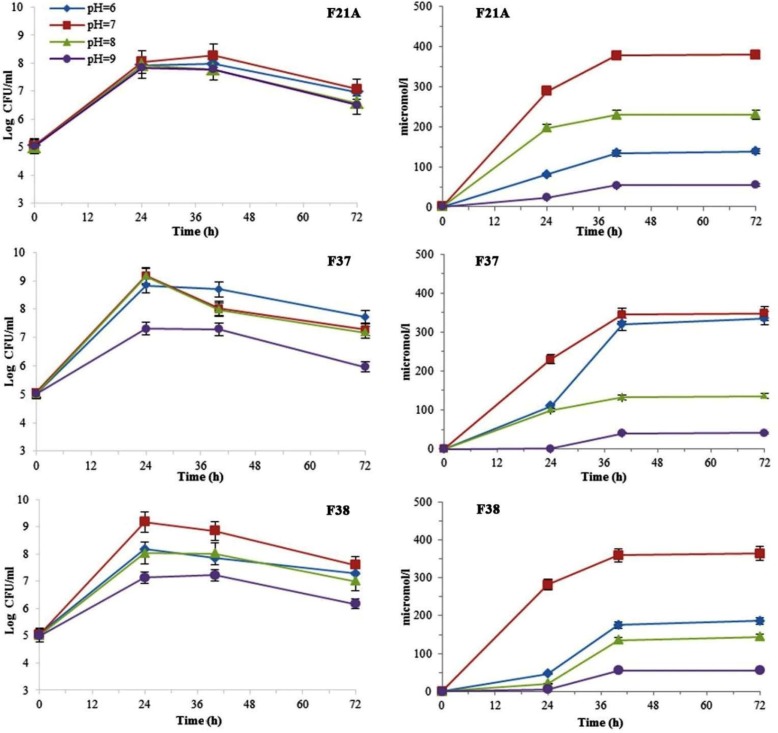
The effect of different initial pH on the bacterial growth kinetics and siderophore production levels. All data presented as mean ± SEM of three replicates.

In solid medium assays, F21A, F37 and F38 were able to growing and siderophore production up to 2.5% salt and 20 to 28°C (data not shown). In liquid medium, in all cases, close associations between growth rates and siderophore excretions were noticeable (Figs. 1 to 3). Generally, the three mentioned isolates all were able to grow at temperatures of 20 to 37°C with the higher growth at 28°C (Fig. 1). When incubated at 37°C, none of the isolates entered exponential growth phase and, consequently, siderophore production was not detectable. Under such conditions, the maximum growth rates for the isolates were reached after 24 h of incubation at 28°C while maximum siderophore production delayed for 40 h and arrived at stationary phase after 72 h at 20°C. Comparatively, the highest siderophore production level was observed for the isolate F21A after 72 h at 20°C (1359.5 μMl^−1^; Fig. 1).

As shown in Fig. 2, reduction of the bacterial growth and siderophore producing values was reversely correlated with NaCl concentration in the medium. Such that, the least rate of growth was detected in 5% NaCl. None of the bacterial strains were able to produce a siderophore in salt concentration above 2.5%. At low salt medium (1%) the maximum level of siderophore production was 242.5 μMl^−1^ at the end of the logarithmic phase for F37 and F38.

Having inoculation into medium with various initial pH values, growth and siderophore excretion of all three isolates were reduced in both high alkaline and acidic media (Fig. 3). The highest siderophore values in acidic or alkaline pH were for F37 (345 μMl^−1^) and F21A (230 μMl^−1^) isolates, respectively (Fig. 3).

Furthermore, increasing amounts of soluble zinc in liquid medium led to elevated siderophore production by the strains, while even low concentrations of iron in broth medium inhibited the siderophore production. Interestingly, at any concentration of soluble zinc, F37 isolate produced higher siderophore amounts compared to the other strains (Table 2).

**Table 2:** Influence of exogenous iron and zinc concentrations on siderophores productions by the selected isolates.

**Bacterial strains**	**Zn concentration (mg l^−1^)**					**Fe concentration (mg l^−1^)**				

	**0**	**75**	**125**	**250**	**500**	**0**	**25**	**50**	**100**	**200**
F21A	200	200	270	370	400	200	ND	ND	ND	ND
F37	300	400	800	850	960	300	ND	ND	ND	ND
F38	295	250	290	400	616	296.6	ND	ND	ND	ND

* Data units are μmoles liter^−1^.

ND, not detectable.

### Quantitative assessments for zinc solubilization

**Fig. 4:**
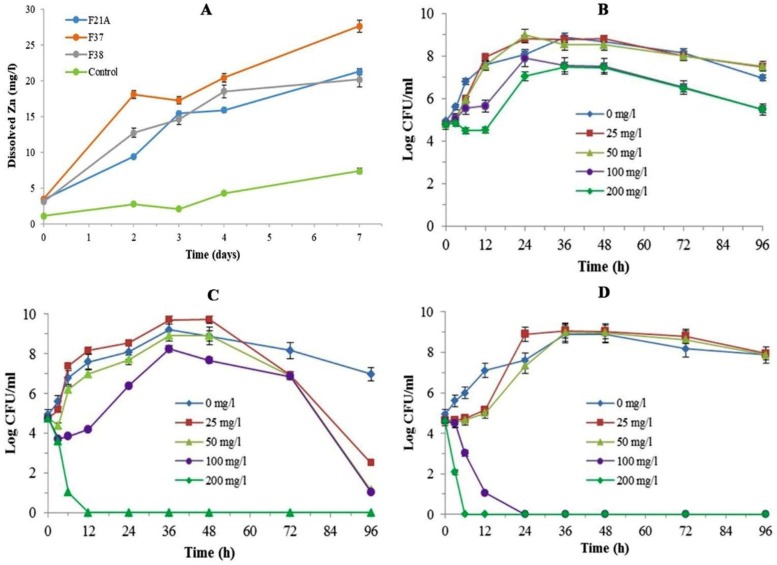
Released soluble zinc level by the selected strains as assayed in the liquid medium (A); Tolerance of the selected strains F21A (B), F37 (C) and F38 (D) to various concentrations of soluble zinc in the broth medium. All data presented as mean ± SEM of three replicates.

As shown in Fig. 4, the highest value of soluble zinc obtained 27.62 ppm, 21.27 ppm, and 20.16 ppm after 7 days for isolates F37, F21A and F38, respectively. All the cultures showed a shift in pH after 7 days of growth from neutral to acidic (data not shown). In assessment of isolates tolerance to soluble zinc, for each of the three strains, the highest propagation levels were recorded at 25 mg l^−1^ of soluble Zn (Fig. 4), while at Zn>50 mg l^−1^, a further reduction in propagation was observed. At 200 mg l^−1^ level, F37 and F38 growth were completely inhibited at the first day, while F21A tolerated this concentration for 96 h very well. In other words, the highest tolerance to dissolved zinc was observed in F21A followed by F37 at the concentrations of 200 and 100 mg l^−1^, respectively. The lowest tolerance was obtained for F38 such that it never entered an exponential growth phase when grown in concentrations higher than 100 mg l^−1^.

### Molecular identification of the selected isolates

The selected isolates were subjected to further characterization by biochemical and biological tests as well as *16S rDNA* sequencing to figure out their taxonomy. Alignments of the *16S rDNA* sequences of F37, F38 and F21A isolates revealed that 99%, 98.8% and 99% similarity with the strain *P. japonica* NBRC 103040^T^ (GenBank Accession Nos. BBIR01000146), respectively.

### PGPR activities of the selected isolates at greenhouse assays on maize

**Fig. 5:**
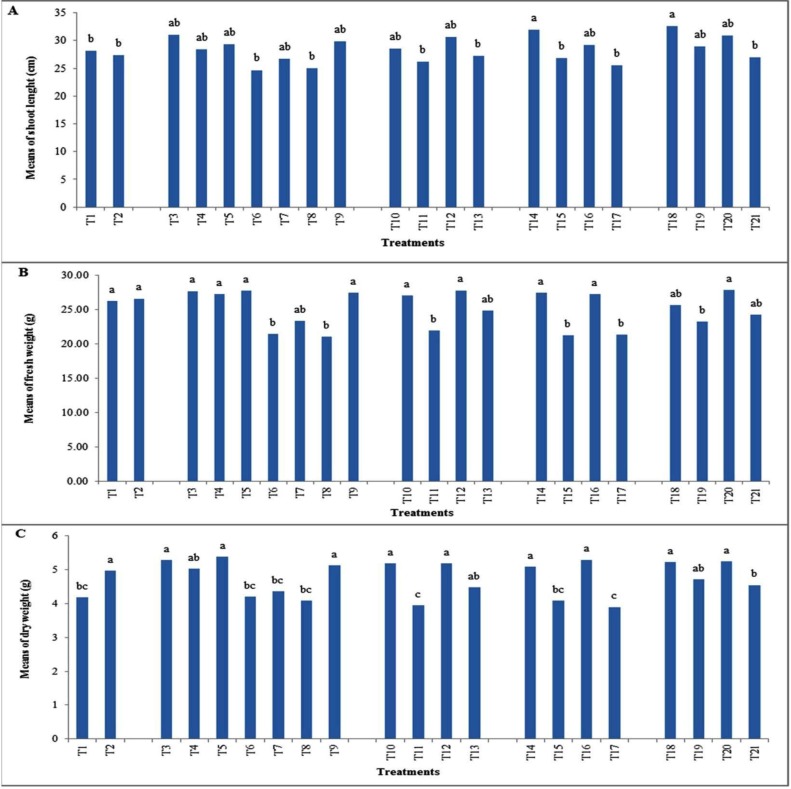
Effect of the isolates on maize growth indicators. Panels show variations in the shoot length (A), fresh weight (B) and dry weight (C) of 60 days old plants grown in greenhouse conditions with treatments described in Material and Method section. Means comparison was done with Duncan’s method. Means with the same letters are ranked in the same groups.

Inoculation of maize seeds with seven selected isolates significantly increased the shoot length and plant biomasses (Table 3 and Fig. 5). Under controlled conditions, plants inoculated with isolates F21A, F37 and Z29 produced the highest shoot height and shoot fresh and dry weight compared to the other treatments. Also, treatments with isolates F21A and F37 in combination with Z29 improved these three indicators. Comparable growth rates between plants receiving chelated Fe and plants inoculated with F21A, F21B, F37 and Z29 isolates were noticeable.

**Table 3:** Analysis of variance of plant height, fresh weight and dry weight of maize inoculated with the selected isolates.

**S.O.V**	**DF**	**Plant height (cm)**	**Fresh weight (g)**	**Dry Weight (g)**
Block	3	2.058^ns^	23.722^ns^	0.698^ns^
Fe+Zn Bacteria	4	4.534^**^	69.646^*^	2.981^*^
Block× Fe+Zn Bacteria	12	47.400^*^	37.690^*^	2.456^*^
Error	60	727.877	628.181	23.749
CV	-	13.06	14.13	13.17

ns, non-significant,

^*^ and ^**^ significant differences at *P*< 0.05 and *P*< 0.01, respectively, compared to the control group.

## DISCUSSION

Diverse groups of rhizobacteria, collectively called plant growth promoting rhizobacteria (PGPR), have been of interest to enhance directly and/or indirectly the growth of plants. One of the most well-known groups of PGPR are siderophore producing bacteria (21, 22). Given that the ability to dissolve of insoluble zinc is not a common feature amongst the soil-borne bacteria (23), the isolation and investigation of siderophore-producing bacteria which simultaneously can effectively zinc solubilization was favored in this research. According to our results and also the previous works in Iran (24, 25), many indigenous bacteria have high ability to produce siderophores. We also identified hydroxamate and carboxylate types of siderophores based on O-CAS manner (7).

Siderophores are secreted under iron-depleted conditions and their production is inhibited by iron due to suppression of the siderophore-related genes expressions (26). We found that great amounts of siderophores were synthesized in lack of iron whereas no siderophore production was observed in >25 μM of iron available to bacteria. Consistently, Gaonkar and Bhosle (2013) reported that Fe^+2^ and Fe^+3^ below 2 and 40 μM concentrations, respectively, induced siderophore production (27). Dave and Dube (28) have reported 27 μM of iron as threshold level, which stopped siderophore production while, in another study, 20 mM of iron repressed siderophore production (29). Our findings showed that, unlike iron, increasing the amount of soluble Zn in the medium could improve the production of siderophore (Table 2) which is in consistent with previous findings (27, 30) showing that elevated concentrations of zinc ions favor the siderophore biosynthesis. This phenomenon emphasizes that siderophore producing isolates with ability of dissolving inorganic zinc compounds could serve as efficient bio-fertilizer to improve Fe and Zn nutrition of crop plants simultaneously. For this purpose, we investigated the potential of Zn solubilization in selected isolates as well. The ZSI data, obtained in the solid and liquid medium in this research (Table 1 and 2), was significantly higher than the other observations (8, 15, 31) indicating that the isolates adapted to alkaline soil conditions are efficient Zn solubilizing bacteria.

Major amounts of solubilized zinc, which were released in the liquid medium, existed as free cationic Zn^2+^ which is readily absorbed by plants. However, plants can tolerate higher level of Zn metal by homeostatic mechanisms (32), but at supraoptimal concentrations, Zn can be toxic and decrease the viability of microorganisms. Therefore, the proper concentration of Zn ions is essential for correct outcome. In the present study, tolerance of the isolates was assessed at various levels of soluble zinc and showed that F21A isolate is well adapted to as high as 200 mg/l of Zn ion. Saravanan et al. (10) previously reported that ZSB-O-1 and ZSB-S-2 isolates were able to tolerate 100 mg/l of Zn in liquid medium.

It is important to find PGPR isolates from native soil which tolerate to various environmental conditions. In this study, all the selected strains tolerated >2.5% of added NaCl (solid medium results) indicating that these isolates would functionally be active in most cultivated lands in which the salinity of soil is usually below 2.5%. Gaonkar and Bhosle (27) isolated strains with low siderophore production in the presence of ≥ 2% NaCl. Decreased growth rates above 37°C for the isolates are accepted as in natural situation these bacteria survive in mid days while continuing to grow in the rest of the day. Our data also suggests that despite reduction of bacterial growth under cold environments conditions, siderophore producing activity of strains is increased. This is favorable for plant which absorb low iron at low temperatures. Based on results of growth rates and siderophore production of bacteria in pH 6 to 9, high siderophore values secreted by F21A isolate at alkaline pH (Fig. 3) advocates its potential for using as bio-fertilizer in agricultural soils of the region which are predominately characterized by high pH (33). Higher pH is also commonly related with the decreased absorption of Zn by plants (34). So, the release of 21.27 ppm zinc ions by F21A isolate can influence Zn availability for plants, which confirm by greenhouse assays.

Four of the selected strain significantly promoted maize growth influencing on three indicates (the shoot length, fresh and dry weights) (Fig. 5). Similarly, significant increasing of plant parameters such as dry matter yield, productive tillers, number of panicles, number of grains, grain yield and straw yield in rice were observed under ZSB inoculation (35). Furthermore, in line with our findings, improvement of maize growth with siderophore producing *Pseudomonas* spp. has been reported (36, 37). Goteti et al. (2013), indicated that bacterial inoculation of maize with zinc solubilizing *Pseudomonas* spp. and *Bacillus* spp. enhanced the plant growth significantly after 60 days (14). In this study, inoculations with the strains F21A, F21B, F37 and Z29 showed a remarkable increase in dry weight means comparable to conditions when 6% iron EDDHA chelate were added (Fig. 5).

## CONCLUSION

In conclusion, we isolated several siderophore-producing strains, three of which (F21A, F37 and F38) demonstrated the highest capability of Zn solubilization and well adapted to various environmental conditions. Our data also indicated that bacterial inoculation in maize leads to a higher yield potential. Biochemical and molecular characteristics were shown that these strains showed high similarity to the strains of *P. japonica*. These findings demonstrated that the potential of *P. japonica* strains as PGPR in iron and zinc deficient soils. Moreover, the *P. japonica* strains F21A, F37 and F38 could be more promising for being used as Fe and Zn bio-fertilizers, in the future.
